# Factors Related to the Intention to Use Dental Care by Industrial Workers Due to COVID-19: Application of Anderson Model and Planned Behavior Theory

**DOI:** 10.3390/ijerph191912883

**Published:** 2022-10-08

**Authors:** Hye-Ran Eun, Jong-Tae Park, Jong-Hwa Jang

**Affiliations:** 1 Department of Oral Health, Graduate School of Health and Welfare, Dankook University, Cheonan-si 31116, Korea; 2 Department of Oral Anatomy, College of Dentistry, Dankook University, Cheonan-si 31116, Korea; 3 Department of Dental Hygiene, College of Health Sciences, Dankook University, Cheonan-si 31116, Korea

**Keywords:** anxiety, COVID-19, dental care, intention, stress, workers

## Abstract

The COVID-19 pandemic has affected medical and dental care in Korea. This study aimed to investigate the factors influencing the intention to use dental care in industrial workers, an economically active population. An online questionnaire survey was completed by 301 industrial workers. Stress and anxiety to viral epidemics-6 (SAVE-6), attitude, subjective norm, perceived behavior control, and intention to use dental care, based on the theory of planned behavior (TPB), were measured. Predisposing, enabling, and need factors based on the Andersen model were measured as control variables for influencing factors. In the final hierarchical multiple regression analysis, the most significant relevant factors affecting intention to use dental care were attitude (β = 0.598, *p* < 0.001), followed by experience of dental clinic visits (β = 0.237, *p* < 0.001), subjective norm (β = 0.125, *p* < 0.001), perceived behavior control (β = 0.114, *p* = 0.004), SAVE-6 (β = −0.073, *p* = 0.025), and gingival bleeding (β = 0.062, *p* = 0.029). Dental care use decreased to 58.5%, and socio-psychological factors based on TPB were closely associated with the intention to use dental care. Therefore, awareness must be raised regarding oral health practices to increase the use of dental care among industrial workers.

## 1. Introduction

Severe acute respiratory syndrome coronavirus-2 (SARS-CoV-2)—the virus that causes coronavirus disease 2019 (COVID-19)—transmits through droplets from the saliva of an infected person [1]. Changes in daily life due to the COVID-19 pandemic led to fear of COVID-19, resulting in people avoiding hospital visits and postponing scheduled visits [2,3,4].

Handpieces or ultrasonic scalers used in dental procedures generate a large amount of aerosol [5,6]. Both the patients and the medical staff are at a higher risk of infection in dentistry due to their exposure to such an environment [7,8,9]. Great concerns exist about the spread of infection owing to the high probability of the spread of droplets from the mouth [10,11], which increased anxiety and stress [12,13], resulting in the avoidance of dental clinic visits and reduced dental clinic utilization [4,14,15,16]. The inability of patients to utilize healthcare services promptly results in poor health conditions [17], which increases their socioeconomic burden [18]. Since most workers were less likely to voluntarily visit a dental clinic for a periodic dental check-up due to their circumstances compared with non-workers, they miss the opportunity to treat oral diseases through early detection [19]. If industrial workers—who play a pivotal role in the economically active population—suffer from chronic oral diseases by avoiding dental care use due to the COVID-19 pandemic, the resultant economic losses are expected to be much greater [20].

Industrial workers, accounting for half of the population, spend most of their time at the workplace, supporting their families as guardians, and acting as a pivotal population of the nation by actively engaging in economic activity [17,21]. Accordingly, relevant factors should be analyzed by closely investigating the changes in dental care use to prepare for COVID-19 and other emerging infectious diseases. Recent studies related to domestic healthcare services use included patients with rheumatoid arthritis [22] and diabetes mellitus [23], but there are no studies investigating the factors affecting industrial workers’ use of dental care services in Korea. It is necessary to thoroughly analyze the factors related to the use of dental care by industrial workers to activate industrial oral health applied to the economically active population.

Andersen’s model (AM) and the theory of planned behavior (TPB) are widely used theoretical models that can identify the factors associated with healthcare services and dental care use [24,25,26]. The models will be useful in analyzing the factors influencing the intention of dental care use among industrial workers during the COVID-19 pandemic [4]. In particular, the stress and anxiety to viral epidemics-6 (SAVE-6) scale is a reliable, valid, and useful scale applicable to the general population [25]. The use of dental care by industrial workers is essential for oral health, even in the context of infectious diseases. Therefore, our research hypothesis was “What are the factors related to the intention to use dental care among industrial workers due to COVID-19?”

This study aimed to investigate the socio-demographic characteristics, level of SAVE-6, and dental care use, to identify the factors affecting the intention to use dental care among industrial workers based on AM and TPB during the COVID-19 pandemic.

## 2. Literature Review

### 2.1. AM and SAVE-6

AM, also known as the “Behavioral Model of Health Service Utilization” [26], is the most common and widely used socio-behavioral behavioral model for health service use factors [24]. It has been used as the main model as it is an integrated model that considers internal and external factors affecting various personal attributes that influence the use of medical services [27]. This has played an important role in providing medical services through the analysis of medical service behavior in several previous studies and suggested a development direction. AM has revised in several stages between 1968 and 2008. According to the biography model, the factors that determine the health service use behavior of an individual are “predisposing factors (PE)”, “enabling factors (EF)”, and “needs factors (NF)”. This model focused on individual characteristics. In contrast, the developed extended model was able to predict the health status due to the use of medical services [28]. In previous studies, dental car use decreased as the household income decreased [29], and a significant relationship was found with the experience of using oral health care according to the level of education and age [22]. In summary, AM was selected for use in this study as it was judged to be useful in identifying and analyzing the factors that affect the intention to use dental care among industrial workers due to the COVID-19 pandemic.

#### 2.1.1. PF

PF refers to the characteristics that an individual possesses before the onset of disease and the individual characteristics that directly or indirectly affect the use of medical services, EF, and NF [30]. It comprises demographic characteristics such as sex, age, race, marital status, and education level, and includes individual values, health beliefs, and attitudes toward medical services [31]. Analysis of the demographic characteristics in previous studies showed that females had a higher level of awareness regarding scaling health insurance benefits than males, and females were more likely to benefit from scaling health insurance than males [32]. In addition, oral health care use increase with age, whereas it decreases as the education level decreases [22].

The purpose of this study is to investigate how personal characteristics affect the industrial workers’ intention to use dental care due to the COVID-19 pandemic, such as sex, age, marital status, and education level.

#### 2.1.2. EF

EF is a factor related to the ability and means to make use of available medical services. It is a factor that promotes or hinders the use of medical services [10]. It comprises factors such as an individual’s economic level or income level, occupational type, private insurance coverage, and residential area [29]. In a previous study, there was a difference in the frequency of toothbrushing three or more times a day depending on the occupational type of industrial workers [23]. In the case of economic activity, the number of private insurance policyholders is higher, and private insurance policyholders receive preventive dental care more frequently than those without insurance. It has been widely used [33]. This study examines the relationship between occupational type, number of private insurances, income level, working period, experience of dental care, and intention to use dental care as factors that promote or hinder the use of dental care among industrial workers during the COVID-19 pandemic.

#### 2.1.3. NF

NF is a factor indicating that an individual needs dental service. It is a physiological and psychological factor related to the presence and level of an individual’s disease that is the direct cause of using medical services [34]. It has been reported that the experience of using oral health care increases due to direct causes, such as the presence of toothache, masticatory discomfort, or pronunciation discomfort [22]. In this study, oral health status, toothache experience, gingival bleeding experience, and periodontal treatment experience were classified as NF to investigate the factors that directly affect the intention to use dental care.

#### 2.1.4. SAVE-6

SAVE-6 is a tool developed by Chung et al. [25] to evaluate the stress and anxiety response to viral infections experienced by the general public. The purpose of this study is to investigate the relationship between the SAVE-6 level of industrial workers and their intention to use dental care during the COVID-19 pandemic.

Thus, in the present study, the following is hypothesized:

**Hypothesis** **1.** **(H1).** *Due to the COVID-19 pandemic, the intention of industrial workers to use dental care will differ according to the demographic characteristics based on AM.*

**Hypothesis** **2.** **(H2).** *Due to the COVID-9 pandemic, there will be a significant negative relationship between SAVE-6 and the intention of industrial workers to use dental care.*

### 2.2. The TPB Model

TPB is a theory extended and supplemented by Ajzen in Fishbein and Ajzen’s (1975) Theory of Reasoned Action (TRA). It determines individual behavioral intentions by classifying them into three categories: individual attitudes, subjective norms, and perceived behavioral control [35]. Attitudes toward behavior, subjective norms, and perceived behavioral control predict behavioral intentions with high accuracy and are useful theories for predicting various behaviors [36]. TPB classifies the intention to perform a behavior as a direct determinant, and the factors determining the intention are attitude, subjective norm, and perceived behavioral control [37].

**Figure 1:**
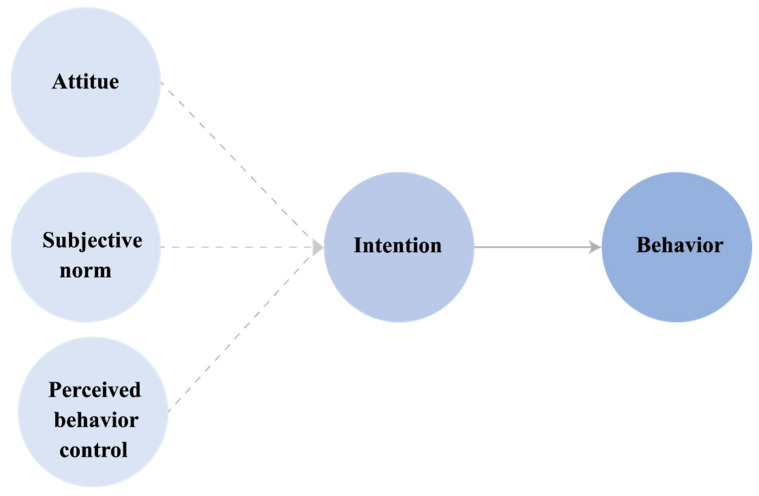
The model of TPB.

Attitude evaluates an individual’s likes or dislikes about behavior, and this study investigated the positive or negative evaluations of the industrial workers’ use of dental care in the context of the COVID-19 pandemic [38]. Subjective norm refers to the social pressure that an individual perceives subjectively when they act. Perceived behavioral control refers to the degree of difficulty or ease with which an individual perceives behavioral performance [38]. TPB has a relatively uncomplicated structure compared with other models [38]. According to the results of previous studies, the intention of children’s oral health behaviors was influenced when they had oral health behavioral intentions in the past; moreover, the attitudes, subjective norms, and perceived behavioral control toward the current oral health behaviors had an effect [16]. The behaviors covered in the study applying TPB include digital content impulse purchase [39], safety behavior [40], career search behavior [41], sexual behavior [42], and drinking and smoking behavior [43]. The TPB model, which has been proven useful in predicting behavioral intentions and performance in studies targeting various behaviors, was applied to this study to understand the intention to use dental care. Figure 1 shows the model of TPB.

Thus, in the present study, we also hypothesized:

**Hypothesis** **3.** **(H3).** *Due to the COVID-9 pandemic, the higher the attitude, subjective norm, and perceived behavioral control level of PBT-based dental care industrial workers, the higher the intention to use dental care.*

**Figure 2:**
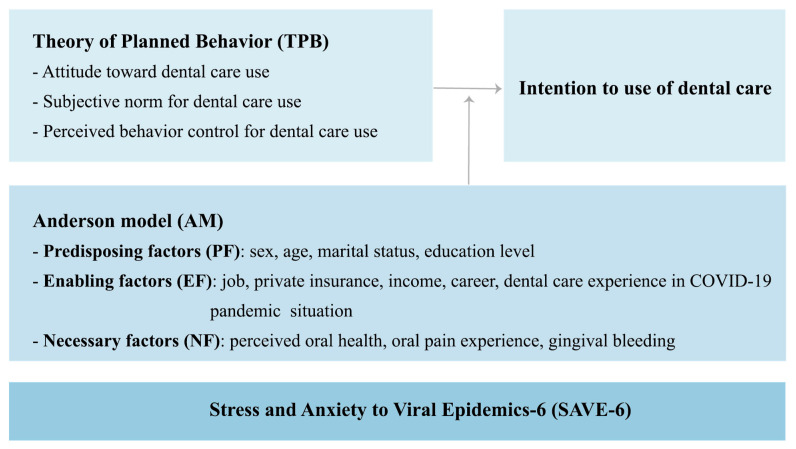
Flowchart of the study.

Figure 2 shows the hypothetical model of this study.

## 3. Materials and Methods

### 3.1. Study Design and Ethical Consideration

This descriptive cross-sectional study analyzed the factors associated with the intention to use dental care among industrial workers during the COVID-19 pandemic by applying AM and TPB.

Based on the principles outlined in the Declaration of Helsinki, all study-related procedures were conducted ethically. Informed consent was obtained from all subjects involved in the study. Moreover, the study was reviewed and approved by the Institutional Review Board of the Dankook University (IRB approval no.: DKU 2022-03-019).

### 3.2. Participants and Data Collection

The population of this study is industrial workers. This study included 301 workers from three industries (Cheonan, Cheongju, and Pohang) located in Korea. The inclusion criteria were:

1. workers currently employed in the industry;
2. those able to complete the questionnaire after fully understanding the study‘s intention and purpose; and
3. voluntarily providing consent to participate.

Incomplete responses—accounting for ≥50% of the questionnaire response rate of the participants—were excluded from the analysis. Using G*Power 3.1.7 (Heinrich Heine University Düsseldorf, Düsseldorf, Germany), the appropriate sample size was calculated to be 298 assuming a significance level of 0.05, with an effect size of 0.1 and power of 0.95, and 16 predictor variables.

A structured online questionnaire survey was created using Naver office (https://office.naver.com/, accessed on 7 March 2022). The participants accessed the URL (https://naver.me/Gc4xmWjE, accessed on 7 March 2022) to complete the survey between 8 March 2022 and 23 April 2022.

The study staff visited the companies and elucidated the purposes and intention of the study to the representatives and managers to receive cooperation and consent. Study participants were recruited via recruitment announcement. Industrial workers who agreed to participate in the study were asked to complete the self-reported questionnaire survey. A total of 302 questionnaires were returned. Among them, one questionnaire with incomplete responses was excluded. Thus, 301 participants were included in the analysis.

### 3.3. Variables

This study classified the participants’ socio-economic characteristics into predisposing, enabling, and need factors based on the AM [22,23]. Based on the TPB, dental care use was classified into the following sub-categories: attitude, subjective norm, perceived behavior control, and intention to use dental care [24]. SAVE-6 was also measured [25].

#### 3.3.1. Control Variables Based on the AM

The socioeconomic characteristics of the participants based on the AM were composed of the following three sub-factors: predisposing factors (sex, age, marital status, and education level); enabling factors (occupation, number of private insurances, income level, working years, and experience of dental care use in the COVID-19 situation); and need factors (perceived subjective dental health, experience of oral pain, and gingival bleeding) [22,23]. The predisposing and enabling factors are categorical variables. The need factors were assessed using the 5-point Likert scale containing five response options: strongly disagree (1 point), disagree (2 points), neither agree nor disagree (3 points), agree (4 points), and strongly agree (5 points). Thus, the higher the mean score, the more the experience and the better the oral health condition.

#### 3.3.2. Use of Dental Care Based on the TPB

The use of dental care based on the TPB in the participants was assessed using 16 questions used in a previous study [24] after its validity was tested by three dental health specialists. Moreover, exploratory factor analysis (EFA) was used to measure three factors (attitude, subjective norms, and perceived behavior control), and the cumulative explanatory power of analysis results was 79.49%. The factors were measured using the 5-point Likert scale containing 5 response options; the higher the score, the more positive the dental care use. Reliability testing of the tool in the previous study revealed Cronbach’s α of 0.997 [24]. Cronbach’s α was 0.945 in the present study.

Intention to use dental care, used as a dependent variable, was measured using four questions with the 5-point Likert scale containing 5 response options. The mean scores were calculated; the higher the score, the higher the intention to use dental care. Cronbach’s α was 0.997 and 0.881 in the previous study and present study, respectively.

#### 3.3.3. SAVE-6

SAVE-6—an instrument that assesses the stress and anxiety of the general population to viral infectious diseases using six questions—developed by Chung et al. (2021) [25] was used in this study. The questions were measured using the 5-point Likert scale containing six response options. The mean scores were calculated; the higher the score, the higher the level of stress and anxiety. In the previous study [25], Cronbach’s α was 0.815, whereas it was 0.835 in this study.

### 3.4. Statistical Analysis

All variables were summarized with descriptive statistics using the SPSS (IBM SPSS Statistics 23.0 for Window, SPSS Inc., Chicago, IL, USA) program, and the data were checked for normality. Differences in intention to use dental care according to the demographic characteristics with an application of AM were analyzed using the independent t-test and one-way analysis of variance (ANOVA). Duncan’s multiple range test (MRT), a post hoc test, was conducted for comparison subsequently. Correlation between attitude, subjective norms, and perceived behavioral control based on the TPB associated with the use of dental care, SAVE-9, and intention to use dental care were analyzed using Pearson’s correlation coefficient. Hierarchical multiple regression has been used frequently in research to predict a dependent variable based on multiple independent variables [44]. Hierarchical multiple regression analysis was performed to investigate the factors associated with the intention to use dental care, and α = 0.05 was considered a significant level.

## 4. Results

### 4.1. Intention of Dental Care Use According to the Socio-Economic Characteristics Based on the AM

Table 1 shows the differences in the intention to use dental care among workers according to the characteristics after classifying them into predisposing, enabling, and need factors based on the AM. In PF, the proportion of intention to use dental care was higher in those who graduated from at least a 4-year college (3.98 points) than in those who graduated from a 2-year or less college (3.61 points) (*p* < 0.001). In EF, the proportion of intention to use dental care was higher in those who visited a dental clinic after COVID-19 (4.14 points) than in those who did not visit (3.30 points) (*p* < 0.001). In NF, the proportion to use intention of dental care was significantly higher in those who had “very severe” gingival bleeding (4.58 points) than in others (*p* < 0.001). There were no differences in the intention to use dental care in terms of sex, age, and marital status in PF; in occupation, the number of private insurances, monthly income, and working years in EF; and perceived subjective oral health and oral pain experience in NF (*p* > 0.05).

### 4.2. Descriptive Statistics on Factors Associated with Dental Care Use and SAVE-6 Based on the TPB

Descriptive statistics on attitude, subjective norms, and perceived behavior control for dental care use, intention to use, and SAVE-6 among industrial workers due to COVID-19 are shown in Table 2. Out of the highest score (5 points), the intention to use dental care score was 3.78 points, the attitude was 3.84, the subjective norm was 3.75, and perceived behavior control was 4.34. SAVE-6 scored 3.07 points.

### 4.3. Correlation between the Intention to Use Dental Care, Attitude, Subjective Norm, Perceived Behavior Control for Dental Care Use, and SAVE-6

Table 3 shows the results of the analysis on the correlation between attitude, subjective norm, perceived behavior control for dental care use, intention to use, and SAVE-6 among industrial workers due to COVID-19. Intention to use dental care positively correlated with attitude (r = 0.769), subjective norm (r = 0.338), and perceived behavior control (r = 0.544), whereas it negatively correlated with SAVE-6 (r = −0.183).

### 4.4. Relevant Factors Affecting Intention to Use Dental Care

Table 4 shows the results of hierarchical multiple regression analysis on the factors associated with the intention to dental care use among industrial workers due to COVID-19.

In Model 1, education level, the experience of dental clinic visits after COVID-19, and experience of gingival bleeding showed significant differences in the intention to use dental care by demographic characteristics and were used as independent variables. In Model 2, SAVE-6 was further added. In Model 3, whether attitude, subjective norm, and perceived behavior control in the TPB significantly affected the intention to use dental care use as independent variables—after variables used in Model 1 and Model 2 were controlled—was examined.

The results of the analysis showed that F was 30.135 (*p* < 0.001), 25.727 (*p* < 0.001), and 99.907 (*p* < 0.001) in Models 1, 2, and 3, respectively, suggesting that all models fit the data well. Moreover, no multicollinearity of the measured variables was confirmed since tolerance (TOL) was ≥0.1 and variance inflation factor (VIF) was ≤2 for all models.

Hierarchical regression analysis was performed on education level, the experience of dental clinic visits after COVID-19, and experience of gingival bleeding in Model 1, which indicated that education level (β = 0.104, *p* = 0.048) and experience of dental clinic visits after COVID-19 (β = 0.104, *p* = 0.048) were significant, while gingival bleeding was not (*p* = 0.076).

When SAVE-6 was further used in Model 2, education level (β = 0.106, *p* = 0.041), experience of dental clinic visits after COVID-19 (β= 0.421, *p* < 0.001), and SAVE-6 (β = −0.158, *p* = 0.002) were significant. Impact on the intention to use dental care increased by 2.3% compared with that of Model 1. Gingival bleeding was not significant in Model 2 (*p* = 0.062).

In Model 3, the effect sizes for influencing factors on intention to use dental care were most significant in the attitude for dental care use (β = 0.598, *p* < 0.001), followed by experience of dental clinic visits after COVID-19 (β = 0.237, *p* < 0.001), subjective norm (β = 0.125, *p* < 0.001), perceived behavior control (β = 0.114, *p* = 0.004), and SAVE-6 (β = −0.158, *p* = 0.002). Gingival bleeding—not significant in Models 1 and 2—was a significant influencing factor (β = adjusted explanatory power of Model 3 was 70.0%. 0.071, *p* = 0.029); however, education level was not a significant influencing factor (*p* = 0.430).

These findings showed that when SAVE-6 increases, the intention to use dental care decreases. Moreover, when oral manifestations such as gingival bleeding got severe, the intention to use dental care increased with high levels of attitude, subjective norm, and perceived behavior control for dental care use.

## 5. Discussion

This study aimed to identify the factors influencing dental care use among industrial workers. There have been several changes in our society due to the COVID-19 pandemic [4,28,29]. As non-human contact (untact) society continues, people’s fear of COVID-19 affected healthcare center [4] and dental care use [45]. Thus, a close analysis of the relevant factors should be conducted.

Since AM and TPB are useful theories for analyzing the factors related to healthcare use [23,24], this study used those theories to examine the factors related to dental care use. The proportion of participants who visited a dental clinic during the COVID-19 pandemic was 58.5%, which was lower when compared with the proportion of Korean adults who used dental care, accounting for 69.5%, based on the data from the 7th Korea National Health and Nutrition Examination Survey (KNHANES) [46]. The increasing rate (9.2%) of medical expenses under national insurance during the first half of 2020—when COVID-19 was spreading—had decreased by 0.3% compared with the average increasing rate (9.5%) for the last 3 years. In addition, a decrease in the number of visit days and people seeking medical care had been reported [3]. This suggests that decreased medical use when infectious diseases, such as COVID-19, emerge is an expected outcome. Moreover, a study reported similar findings that the proportion of people seeking medical care was reduced because patients’ hospital use decreased, and hospitals limited the provision of healthcare services during the SARS pandemic in 2003 [47]. Since dental care institutes are not safe from the phenomenon of avoiding hospital visits, the factors affecting dental care use should be examined and the resultant negative impacts should be prevented in advance.

Jang and Lee [48] reported that the proportion of dental care use among Korean industrial workers increased when the individuals were older, had a spouse, or had a long year of work. Kim et al. [49] reported that factors affecting unmet healthcare needs were sex, age, education level, marital status, household income level, economic activity, type of medical insurance, and subjective health condition. However, this study did not show the relationships between the intention to use dental care and age, marital status, work type, number of private insurances, monthly income, the working year, perceived oral health level, or subjective oral pain. We believe that this resulted from the unique environmental factor, the COVID-19 pandemic.

Attitude, subjective norm, and perceived behavior control for dental care use based on the TPB among industrial workers were measured using the 5-point Likert scale, and all three sub-categories scored ≥ 3.7 points. This finding is a positive response to dental care use in the COVID-19 pandemic, but it was the opposite result of decreased healthcare use reported in previous studies [20,21]. This may be caused by the decreased negative response to dental care use as countermeasures against the prolonged duration of COVID-19 are progressing, and fear and alarm to countermeasures against the infectious disease become desensitized. Moreover, it can be inferred from the results showing an average of 3.07/5 points of SAVE-6.

In the final model, through hierarchical multiple regression analysis, the predisposing factors of AM (experience of visiting a dental clinic during the COVID-19 pandemic), gingival bleeding in the need factors, SAVE-6, TPB-based attitude, subjective norm, and perceived behavior control were significant influencing factors. We were able to confirm that TPB-based perceived oral health during COVID-19 acts as a significant relevant factor in the intention to use dental care. In particular, the intention to use dental care increased as the participants’ gingival bleeding became severe in the present study. This finding is similar to the result of the study by Lee et al. [16], which reported that oral symptoms were a significant influencing factor in oral health-related quality of life and oral health practice was an indirect influencing factor.

The result demonstrating that experience of dental care visits was a significant influencing factor on the intention to use dental care was similar to the result of the study by Choi [50], which reported that periodic dental check-up increases industrial workers’ motivation for preventing and treating oral diseases since they can correctly assess their oral condition. Periodic dental clinic visits are related to periodontal health knowledge, and a specific intervention to promote individualized patient education can positively affect the knowledge of the general population about periodontal health [51]. Therefore, a group oral health management system should be systemically operated in the workplace for oral health workers. Moreover, the decreased dental care use during the COVID-19 pandemic should be considered [52,53]. Since oral diseases are preventable, unlike some other diseases, temporal and economic costs can be reduced if early detection and treatment are implemented [24].

### 5.1. Theoretical and Practical Implications

Health care use declined by about a third during the pandemic, with significant variations and a greater decline among those with less severe illnesses. Addressing unmet needs is a priority; however, research into the health impacts of reduction could help the healthcare system reduce unnecessary care in the post-pandemic recovery [4]. Oral health education can help workers change their perception, knowledge, attitude, and behavior toward oral health, and industries can be accessible and effective places to provide adult oral health education [54]. Thus, to ensure that industrial oral health programs are more active, oral health education should be provided to spread awareness regarding proper oral health practices for workers’ oral health [55].

The results of this study suggest implementing the following measures to promote oral dental health among industrial workers. First, a more multifaceted study should be conducted to identify the level of dental care use and factors influencing the workers in the industry. Second, industrial workers’ intention to use dental care decreased as the SAVE-6 score increased, whereas their intention to use dental care increased as TPB increased. These results indicate that additional support is needed to reduce unmet dental care during an infectious disease epidemic such as the COVID-19 pandemic. In particular, the use of dental care by participants in this study was low (58.5%). This suggests that unmet dental care for adults is related to nondental checkups [18], suggesting that it is necessary to prepare a strategy to activate dental checkups in the workplace. Lastly, multi-modal studies and programs should be developed to promote oral health among industrial workers.

This is the first study to verify that TPB-based attitude, subjective norm, and perceived behavior control for dental care use significantly affected the intention to use dental care among Korean industrial workers during the COVID-19 pandemic. Furthermore, in this study, predisposing, enabling, and need factors of the industrial workers were closely analyzed. Thus, this study is significant.

### 5.2. Limitations and Future Research

Since this study is a cross-sectional survey that included a limited number of industrial workers, it has some limitations. First, this study investigated the demographic characteristics and oral health behavior among industrial workers; however, detailed information about COVID-19—such as vaccination status, self-quarantine following close contact, and history of COVID-19 infection—was not investigated. Moreover, since oral health condition was not measured, we could not analyze the association between information written on the questionnaire survey by workers and their actual oral health condition. Second, since there were very few relevant previous studies, it was difficult to analyze the results of this study for comparison. Third, since 66.2% of the participants were below 30 years of age, the participation rate of middle-aged workers was low because the online questionnaire survey is not familiar to them. Opinions of all age groups were not used evenly. Fourth, in our study, there may be differences from factors affecting actual dental care use, because the factors affecting dental care use intention, which were dependent variables, were analyzed based on AM and TPB in industrial workers. Lastly, structural equation model (SEM) analysis using AMOS or PLS software has been widely used in recent years to analyze the relationship between psychosocial variables [56,57,58,59,60]. Since this study used hierarchical regression analysis, it is limited by the slight difference that may be present from the SEM analysis results, including errors by variable.

In future studies, it is necessary to use an analysis method based on SEM or PLS. In addition, participants of all ages, detailed information about emerging infectious diseases, and dental health check-ups that can study the association with oral health conditions should be included. In-depth studies should be conducted, such as longitudinal studies of follow-up and interventional studies that can reveal causality between each factor and dental care use. We also propose that an evidence-based theory derived from multi-modal studies should be reflected to increase the rate of dental care use in industrial workers, and basic data should be prepared so that industrial oral health programs can be activated for proper oral health promotion behavior.

## 6. Conclusions

During the COVID-19 pandemic, the use of dental care by industrial workers decreased to 59.5%. The intention to use dental care among industrial workers increased as attitude, subjective norm, and perceived behavior control for dental care use based on the TPB were higher. Furthermore, the experience of dental clinic visits after COVID-19 and gingival bleeding was shown to affect the intention of dental care use. Future research should identify the factors related to the use of dental care and make a more practical contribution to patient management. In addition, awareness of proper oral health practice should be raised by oral health education for oral health promotion among industrial workers during the pandemic, and industrial oral health programs should be activated so that dental care use can be implemented periodically.

## Figures and Tables

**Table 1:** Intention to use dental care according to the Anderson model among industrial workers.

Factors	Variables	Division	*n* (%)	Mean ± SD	t or F (*p*-Value)
PF	Sex	Male	167 (55.5)	3.82 ± 0.86	0.717 (0.474)
		Female	134 (44.5)	3.74 ± 0.94	
	Age (y)	19–29	198 (66.2)	3.77 ± 0.89	−0.736 (0.462)
		30–65	101 (33.8)	3.85 ± 0.90	
	Marital status	Married	81 (26.6)	3.73 ± 0.93	0.753 (0.472)
		Single	211 (70.1)	3.79 ± 0.89	
		Others	9 (3.0)	4.11 ± 0.73	
	Education level	College or less	162 (54.0)	3.61 ± 0.88	−3.566 (<0.001)
		University or higher	138 (46.0)	3.98 ± 0.88	
EF	Job	Productive	52 (17.4)	3.70 ± 0.94	1.417 (0.238)
		Office	118 (39.4)	3.88 ± 0.89	
		Research	20 (6.7)	3.96 ± 0.95	
		Others	109 (36.5)	3.68 ± 0.88	
	Private insurance	0	195 (65.0)	3.80 ± 0.92	1.739 (0.159)
		1	89 (29.7)	3.81 ± 0.87	
		2	12 (4.0)	3.25 ± 0.76	
		≥3	4 (1.3)	4.19 ± 0.55	
	Monthly income (10,000 won)	<200	33 (11.0)	3.76 ± 0.89	0.968 (0.437)
		200–250	81 (27.0)	3.66 ± 0.92	
		>250–300	62 (20.7)	3.83 ± 0.84	
		>300–350	55 (18.3)	3.72 ± 0.93	
		>350–400	27 (9.0)	4.03 ± 0.79	
		>400	42 (14.0)	3.91 ± 0.94	
	Career (y)	1–5	202 (67.1)	3.79 ± 0.87	0.094 (0.910)
		6–10	62 (20.6)	3.74 ± 1.03	
		>10	37 (12.3)	3.79 ± 0.80	
	DVE in COVID-19 situation	Yes	175 (58.5)	4.14 ± 0.72	8.976 (<0.001)
		No	124 (41.5)	3.30 ± 0.89	
NF	Perceived oral health	Not at all	11 (3.7)	3.55 ± 1.11	1.108 (0.353)
		Disagree	52 (17.3)	3.78 ± 0.83	
		Neutral	156 (52.0)	3.98 ± 0.92	
		Agree	59 (19.7)	3.73 ± 0.80	
		Strongly agree	22 (7.3)	4.14 ± 0.96	
	Oral pain experience	Very low	19 (6.3)	3.51 ± 1.06	2.128 (0.077)
		Low	93 (30.9)	3.69 ± 0.86	
		Moderate	83 (27.6)	3.70 ± 0.84	
		High	95 (31.6)	3.98 ± 0.83	
		Very high	11 (3.6)	3.93 ± 1.55	
	Gingival bleeding	Very low	35 (11.6)	3.85 ± 0.95 ^a^	5.388 (<0.001) a < b
		Disagree	91 (30.2)	3.64 ± 0.87 ^a^	
		Moderate	75 (24.9)	3.59 ± 0.83 ^a^	
		High	84 (27.9)	3.92 ± 0.92 ^a^	
		Very high	16 (5.3)	4.58 ± 0.62 ^b^	

*p*-value was derived using the independent *t*-test or ANOVA test; ^a,b^ means followed by different letters are statistically significant difference at α = 0.05. SD, standard deviation; PF, predisposing factors; EF, enabling factors; NF, necessary factors; DVE, dental visit experience.

**Table 2:** Descriptive statistics on factors related to dental care use and SAVE-6 based on the TPB.

Variables	Min	Max	Mean ± SD	Cronbach’s α
Intention to use DC	1.00	5.00	3.78 ± 0.90	0.881
Dental care use based on PBT				
Attitude	1.00	5.00	3.84 ± 0.87	0.964
Subjective norm	1.00	5.00	3.75 ± 0.81	0.704
Perceived behavior control	1.00	5.00	4.35 ± 0.69	0.791
SAVE-6	1.00	5.00	3.07 ± 0.85	0.835

Min, minimum; Max, maximum; SD, standard deviation; DC, dental care; TPB, theory of planned behavior; SAVE, stress and anxiety to viral epidemics.

**Table 3:** Correlation between dental care use attitude, subjective norm, perceived behavioral control, intention to use and SAVE-6.

Variables	1	2	3	4	5
Intention to use dental care	1				
Attitude toward dental care use	0.769 **	1			
Subjective norm for dental care use	0.338 **	0.223 **	1		
Perceived behavior control for dental care use	0.544 **	0.556 **	0.270 **	1	
SAVE-6	−0.183 **	−0.135 *	−0.011	−0.115 *	1

* *p* < 0.05, ** *p* < 0.01 by Pearson’s correlation analysis; SAVE, stress and anxiety to viral epidemics.

**Table 4:** Hierarchical multiple regression analysis of related factors affecting industrial workers’ intention to use dental care.

Factor		Variables	Model 1			Model 2			Model 3		
			β	t	*p*-Value	β	t	*p*-Value	β	t	*p*-Value
AM	PF	University or higher (Reference = College or less)	0.104	1.990	0.048	0.106	2.056	0.041	0.026	0.791	0.430
	EF	DVE after COVID-19 (reference = non-experienced)	0.431	8.173	<0.001	0.421	8.084	<0.001	0.237	6.916	<0.001
	NF	Gingival bleeding	0.092	1.780	0.076	0.095	1.872	0.062	0.071	2.199	0.029
		SAVE-6				−0.158	−3.130	0.002	−0.073	−2.254	0.025
		Attitudes for dental care use							0.598	15.236	<0.001
TPB		Subjective norms for dental care use							0.125	3.738	<0.001
		Perceived behavioral control for dental care use							0.114	2.940	0.004
F (*p*-Value)			30.135 (<0.001)			25.727 (<0.001)			99.907 (<0.001)		
R^2^			0.235			0.260			0.707		
Adj. R^2^			0.227			0.250			0.700		

*p*-value was derived using hierarchical multiple regression analysis at α = 0.05; AM, Anderson Model; PF, predisposing factors; EF, enabling factors; NF, necessary factors; DVE, dental visit experience; SAVE, stress and anxiety to viral epidemics; TPB, theory of planned behavior.

## Data Availability

The data presented in this study are available on reasonable request from the corresponding author.
